# Learning everyday multitasking activities—An online survey about people’s experiences and opinions

**DOI:** 10.1371/journal.pone.0312749

**Published:** 2024-12-27

**Authors:** Aina Digaeva, Daniel T. Bishop, Andre J. Szameitat

**Affiliations:** Department of Life Sciences, Centre for Clinical and Cognitive Neuroscience, Brunel University London, Kingston Lane, Uxbridge, Middlesex, United Kingdom; COMSATS University Islamabad, PAKISTAN

## Abstract

Multitasking (MT)–performing more than one task at a time–has become ubiquitous in everyday life. Understanding of how MT is learned could enable optimizing learning regimes for tasks and occupations that necessitate frequent MT. Previous research has distinguished between MT learning regimes in which all tasks are learned in parallel, single-task (ST) learning regimes in which all tasks are learned individually, and mixed learning regimes (Mix) in which MT and ST regimes are mixed. Research using simple laboratory tasks has consistently shown that MT regimes are the most efficient–the so-called *dual-task practice advantage*. However, it is currently unclear which learning regimes are used in everyday life, and which regime people would prefer if given a choice. To answer these questions, 72 participants completed an online survey to describe their real-life experiences of MT learning (e.g., when learning to drive), their opinions about learning MT activities, and filled out the Multitasking Preference Inventory to assess polychronicity. Descriptive statistics showed that for everyday activities, particularly learning to drive, Mix regimes were both the most used and most preferred method, whereas MT regimes were the least preferred. A potential explanation is that everyday MT tasks are typically complex, and so people prefer to learn the individual tasks first, before combining the tasks into an MT learning regime. Preference to engage in MT, as assessed by the MPI, positively correlated (Pearson’s *r* = .24) with preference for MT learning regimes, suggesting that individual differences in learning of complex everyday MT activities can be determined. In conclusion, everyday life multitasking activities such as learning to drive are mostly learned in Mix regimes, i.e. a combination of ST and MT training, and people’s preference to learn such activities with MT regimes increases with their level of polychronicity.

## 1. Introduction

### 1.1 Multitasking

Multitasking (MT) is defined as the performance of more than one task at a time [1]. The pace of everyday life is speeding up with the development of technology such as wireless connectivity and in-vehicle smartphone interactivity, which increases MT propensity [2]. Although doing several tasks at once may seem convenient, research has shown that MT often creates so-called MT costs in the form of increased error rates and/or response times when compared to single-task performance [3,4]. These MT costs have also been shown in applied contexts including studying [5–7], driving [8,9], and office work [10].

Despite these MT costs, there are many occupations for which MT is an essential capability, such as certain medical and military jobs surgeons, nurses, and air-traffic controllers [11,12]. MT costs can be critical, because even a half -second delay in responding, or potential mistakes in tasks, could lead to life-threatening situations–not only in specialised occupations, such as air traffic controllers and surgeons, but also in everyday activities characterized by MT, such as driving. Moreover, efficient MT is required by many employers that maintain high-pressure workplaces, where employees are required to juggle several high-priority tasks at the same time [13]. Clearly, MT is an integral aspect of many people’s lives.

Because of the prevalence of MT activities in everyday life, it would be advantageous to gain an insight into how they are learned. The importance of learning MT was noted by Russ and Crews in their examination of MT behaviours in organisations [14]. Their findings revealed that, although MT was commonly required, only a few organisations provided MT training. And importantly, younger employees consistently overestimated their MT abilities. Learning how to multitask may equip employees with a clearer understanding of the level of MT performance required to perform optimally within their roles and may also enable more realistic self-perceptions of their capabilities [15].

### 1.2 Multitasking learning

Learning MT has attracted the attention of researchers in various fields, including behavioural psychology [5,16–21] to neuroscience [22,23]. In addition, applied studies investigated MT learning of laparoscopic skills in medical students [12,24].

Commonly, MT learning studies compare two different learning approaches. For example, Bender et al. [16] compared two groups of participants, both of which were trained in MT over a series of six sessions, under different learning regimes: One group practised the tasks separately one after another as single tasks (ST), an ST learning regime. The other group practised both tasks simultaneously (dual-task, DT), i.e., an MT learning regime. The authors did not measure participants’ overall task performance, but instead assessed the relative MT costs–specifically, the error rates and response times in the MT regime as compared to the ST regime. Bender et al. concluded that only the MT regime group showed a reduction in MT costs [16]. This means they learned to multitask, while the single-task regime group only showed moderate cost reductions, which were most likely caused by learning the individual tasks more effectively. The greater reduction in MT costs for the MT-regime relative to the ST-regime has been called the *dual-task practice advantage* (DTPA). An extensive review of empirical evidence for the DTPA was presented by Strobach, who also discussed underlying cognitive mechanisms of MT learning and described the DTPA in relation to real life problems, such as how MT learning could transfer to real-world contexts [25].

For the present manuscript, we define three different learning regimes. In (1) single-task (ST) and (2) multitasking (MT) regimes, participants practice only (1) the single-task or (2) the multitask, respectively, for 100% of the time. (3) Mix regimes are defined as training regimes where participants practice ST as well as MT to a notable amount. Such mixing of training regimes could take various forms, for example first only practicing ST for a while and then only MT or going back and forth between ST and MT training. Note that the vast majority of previous studies using the term “MT training” actually used a Mix training regime according to our definition, because virtually all previous studies mixed ST and MT training and compared it with pure ST training to show the DTPA (e.g., [16,26]).

MT is omnipresent in everyday life in a variety of tasks, and there is a need to optimise how to learn multitasking. However, virtually everything we know about MT learning comes from lab-based research using basic cognitive tasks, and it is unclear whether the findings from such tasks, e.g., those relating to the DTPA, generalise to complex everyday tasks. Strobach suggested that there is a possibility that the same mechanisms involved in the DTPA in laboratory tasks may also be involved in real-life MT with very demanding and unpredictable situations [25].

### 1.3 Attitudes towards multitasking

People’s attitudes towards a desired behaviour strongly influence their intention to engage in behaviour, and whether they ultimately perform it [27,28].

Consequently, an individual’s attitudes towards MT may affect their MT behaviour, including their learning regime preferences. To assess whether people like to engage in MT, Slocombe & Bluedorn introduced the term “Polychronicity”, which they defined as one’s preference to engage in MT as a lifestyle, as opposed to completing tasks individually. To measure polychronicity they designed the “Multitasking Preference Inventory” (MPI) [29]. In the MPI, participants are asked to agree or disagree on a four-point Likert scale to 14 statements relating to whether they prefer single- or multitasking (e.g. “I have a one-track mind” or “I do not like having to shift my attention between multiple tasks”), resulting in a single score where higher scores reflect higher polychronicity. It is conceivable that people with a high MPI score might prefer MT learning regimes, and that people with a low MPI score might prefer ST learning regimes.

Since MT is omnipresent in our daily lives, it is evident that people ultimately manage to learn how to do it. However, it is not clear *how* people learn multitasking activities. The primary aim of the current study was to better understand how MT activities are learned in everyday life, by asking participants about their experiences of MT; for example, the kinds of MT activities they learned, the regimes they used, and whether they were satisfied with the outcomes of those regimes. A second aim of this study was to explore the relationships between MPI scores and participants’ expressed preferences regarding learning regimes. For this, we tested for associations between the MPI data and the preferred learning regimes.

## 2 Method

To assess participants’ experiences and opinions about how they learnt multitasking activities in everyday life we developed a new survey consisting of a mix of multiple-choice and open textbox answers. The survey consisted of four parts, starting with informed consent and demographic information. In the second part, assessing their experience, the concept of multitasking was explained, and participants were asked in detail about their experience of one activity (most participants chose learning to drive). In the third part, the MPI was presented. Finally, in the fourth part participants were asked about their opinions on MT, and how they would prefer to learn complex everyday activities.

Participants had to be 18 years or older to participate and the study was approved by the College of Health, Medicine and Life Sciences Research Ethics Committee at Brunel University London.

### 2.1 Participants

Seventy-two participants– 49 men (M = 29 yrs; SD = 7.09 yrs; 18–44 yrs) and 23 women (M = 32.4 yrs; SD = 7.58 yrs; 18–44 yrs)–were recruited online via Testable Minds (https://www.testable.org), an online platform where participants can register to participate in experiments and surveys for payment. Survey completion times ranged from 20 to 45 minutes. The data collection period was from 03 November 2021 until 26 November 2021. All participants gave their informed consent before taking part and each of them received £3 for completing the survey.

### 2.2 Materials and procedure

To our knowledge there are no existing questionnaires which assess the experiences and preferences of learning multitasking abilities. Therefore, the current survey was developed by the authors (except for the MPI) and was guided by the key research questions.

The online survey was presented via the Qualtrics (Provo, UT) online survey platform. The survey consisted of four blocks. In the first block, participants were presented with an information sheet and the consent form. Once they had given their consent, participants read instructions that provided definitions of MT and learning regimes, and examples of MT activities. Thereafter, participants provided demographic information including their age, gender, occupation, driving license status, and self-reported MT ability. The second block comprised of questions about their MT experiences including how they learned them, for example whether they learned in a manual or automatic vehicle, whether there was an instructor present, and, if so, whether the instructor provided explicit MT instructions. However, if someone indicated that they had not learned to drive, they would be redirected to a response box in which they could provide another example of an MT learning experience. The third block included the MPI questionnaire. Lastly, the fourth block included questions about participants’ experiences and attitudes regarding MT learning. This block was designed to gather their opinions on the best approaches for learning to multitask, as well as open-ended questions about factors that had either facilitated or hindered their learning in such contexts, whether current MT learning practices could be improved, and whether participants would have benefitted from training focused on MT. All the personal information of participants was strictly confidential, the collected data was anonymised by Testable Minds.

### 2.3 Data analysis

Some of the data are descriptive in nature (e.g., the percentage of participants preferring a certain learning regime) and are therefore presented with their means and standard deviations. Other data are qualitative in nature (such as responses to most open-ended questions, e.g., ’How to best learn multitasking activities’) and were analyzed for common themes. If common themes were identified (e.g. “It’s important to first learn the tasks by themselves before switching to perform them as multitask”), the number of participants who stated such themes are presented. Finally, for the question whether polychronicity is associated with the preferred learning regimes, Pearson’s correlations were calculated.

## 3 Results

### 3.1 Experiences of learning real-life multitasking activities

Of the 72 participants, 14 participants indicated a single-task activity or no activity at all when asked about their previous experience of learning an MT activity (Table 1). Therefore, these 14 participants were excluded for the analyses in this section, i.e., regarding their experiences in MT learning.

**Table 1:** MT and Non-MT learning activities identified by participants (n = number of times mentioned).

Multitasking Activities	n	Non-Multitasking Activities*	n
Driving	34	Office job	3
Videogames	9	Studying	3
Cooking	6	Archery	1
Playing musical instruments	4	Cricket	1
Working on two computers/ computer applications	3	Preparing Power Point presentation	2
Cycling	2	Sewing	2
**Total:**	**58**	**Total:**	**12**

Note. Non-MT activities were excluded from the subsequent analysis, as were 2 non-responses.

The results indicate that 43% (25 out of 58) of the participants had learned their MT activity in a mix learning regime; 33% (21 out of 58) in a single-task (ST regime) and 24% (12 out of 58) in an MT (MT regime). This shows that all major variants of how MT activities can be learned seem to be practised to some extent in everyday life.

Participants were asked to describe their learning experiences in more detail via open response boxes, but many participants did not do so. Of the 25 (43%) participants who said they learned the MT activity in a mix regime, 6 provided comments. For example, Participant 49 stated, “Because driving a car is a practical way, I used to learn each activity first and then drive around only on the training grounds until I was comfortable to drive on the road with normal traffic”, and Participant 68 stated “For me learning to drive a car was very challenging as it was difficult to remember all the instructions and follow the sequence, for example, remembering to check the blind spots before you manoeuvre and clutch and gear sequence.”. The majority of the comments acknowledged the demands on their concentration and ability to remember the sequencing of the tasks.

Of the 21 (33%) participants who said they learned the MT activity in a ST regime, 7 provided comments. Seven participants described mastering tasks individually; examples included “I learned driving or any other skill by doing one thing at a time. I cannot do all together right away or mixture of this because that confuses and frustrates me” (Participant 6), and “I started to learn step by step by practising it day by day” (Participant 32).

Finally, of the 12 (24%) participants who said they learned the MT activity in an MT regime, 3 provided comments. Examples of comments included “Learning to drive was easy. Some people have hard time concentrating on both gear shift and steering control, but it was easy for me to concentrate on both” (Participant 2), “I think that I multitask a lot of times in my daily life, like listening to a podcast while working. Also, it seemed quite easy to learn to drive, I didn’t feel like I was multitasking.” (Participant 18) and “I multitask when playing games. I have to constantly be listening to what my friends are saying on the mic while using my hands on the controller and playing the game” (Participant 54). The comments suggest that these participants found it easy to perform MT learning from the outset. However, their phrasing left an interesting question open, namely whether they learned in an MT regime because MT is generally easier for them, so that they more frequently engage in it, and/or whether they benefited from prior MT practice which transferred to current tasks.

Of the 58 participants who reported learning an MT activity, 67% (39 out of 58) learned with an instructor and 33% (19 out of 58) learned an MT activity without one. Eighty-two percent (32 out of 39) of the former indicated that the instructor has given them explicit MT instructions, whereas the remaining 18% (7 out 39) stated that their instructors did not teach how to perform or manage MT aspects of the activity. In another question, 85% (33 out of 39) of participants liked the instructor’s approach to teaching the MT aspects of the activity, and only 15% (6 out of 39) of participants would have preferred a different approach. Therefore, most instructors seem to explicitly teach how to manage multiple tasks when teaching MT activities, and the learners were mostly satisfied with this approach.

### 3.2 Opinions on learning multitasking activities

Participants were asked to select statements indicating their agreement with a series of opinions regarding the best approaches to MT learning; multiple answers were allowed. The statement “I prefer to first learn each task on its own until I master them all, and only then train them together” was selected by the majority (65%; 47 out of 72) of participants, which indicates that they preferred a mix learning regime. Only 25% (18 out of 72) reported preferring an MT regime by selecting the statement “I prefer to start right away learning all tasks together”. Fifty-four percent (39 out of 72) of participants agreed with “I think the best strategy depends on the nature of the task”, which indicates that the learning regime preference would be task-specific. Additionally, 18% (13 out of 72) of participants agreed with the statement “it is useful if instructors/teachers explicitly teach how to integrate the tasks (in other words, they teach how to multitask)”.

Forty-six percent (33 out of 72) of participants provided additional comments on how to best learn MT activities. These comments were categorised into recurring themes (see Table 2). For example, “…yes, because I can’t just jump into a task that I have no knowledge of, I have to first familiarize myself with each task then later I could multitask to get better results” and “I personally prefer to accomplish one task perfectly before moving onto the next task” were organised into the theme *First*, *familiarising and/or mastering individual tasks*. Thirty-five per cent (25 out of 72) of all participants provided responses within this theme.

**Table 2:** Recurring themes in comments regarding the statement: “How to best learn multitasking activities".

Themes	%	N
First, familiarising and/or mastering individual tasks until performance is improved, then MT	35	25
Improving one’s levels of focus and attention towards practicing MT	4	3
Belief in oneself and confidence boost from the progress of learning	4	3
Improving one’s timing and planning to be efficient at MT	3	2

Note. 54% (39 out of 72 participants) did not provide any comments.

Next, participants were asked to indicate the extent of their agreement, on a 5-point Likert scale, to the question “learning an MT activity differs from learning a single-task activity”. Of 72 participants, 43% (N = 31) strongly agreed and 46% (N = 33) somewhat agreed with the statement. Just 7% (5) reported that they neither agreed nor disagreed with the statement, and only 4% (N = 3) reported that they somewhat disagreed, and no participants strongly disagreed with the statement. Therefore, the majority of the participants believe that learning MT and ST activities differs.

For the statement “Learning an MT activity would benefit from a learning strategy which takes the MT aspects explicitly into account.” (1–5 Likert scale), 36% (26 out of 72) of participants reported that they strongly agreed, and 56% (40 out of 72) somewhat agreed. Only 5% (4 out of 72) reported that they neither agreed nor disagreed, and only 3% (2 out of 72) reported that they somewhat disagreed with the statement. Therefore, most participants indicated that there is a need for MT-specific strategies in learning.

For the statement “that the current practices of learning MT activities could be improved” 44% (29 out of 72) of participants reported that they strongly agreed, and 38% (27 out of 72) somewhat agreed. Also, 17% (12 out of 72) participants neither agreed nor disagreed, 10% (7 out of 72) indicated they somewhat disagree and only 1% (1 out of 72) strongly disagreed with the statement. Therefore, the majority of participants agreed that current practices of learning MT activities require improvement.

Additionally, participants were asked whether they might benefit from a generic MT training. Overall, 79% (57 out of 72) of the participants reported that they might benefit from a generic MT training, and 21% (15 out of 72) did not believe that MT training would benefit them.

#### 3.2.1 MPI questionnaire

The mean MPI score was -0.18 (SD = 0.826, scale -2 to +2), which indicated no strong preference regarding MT. A significant positive correlation (*r* = .24; *p* = .04) was found between MPI scores and participants’ agreement ratings for the statement “I prefer to start right away learning all tasks together”, which indicates preference for MT learning regimes (Table 3). The correlation assessing the relationship between the average MPI scores and the statement indicating preference for ST learning regime: “I prefer to first learn each task on its own until I master them all”, was negative (*r* = -.19), i.e., the lower the expressed preference to engage in MT, the higher the preference for ST learning, but this failed to reach statistical significance (*p* = .113).

**Table 3:** Correlations of MPI scores with participants’ responses to selected survey items.

Item	MPI-Score	
	Pearson’s *r*	*p*-value
*I prefer to start right away learning all tasks together*	0.242*	0.041
*I prefer to first learn each task on its own until I master them all*	-0.189	0.113
*It is useful if instructor teaches explicitly MT aspect of the activity*	-0.117	0.327
*The best strategy depends on the nature of the task*	0.010	0.933
*Learning in MT regime differs from learning in ST regime*	0.214*	0.035
*I would benefit from a generic MT training*	-0.031	0.794

Note. * *p* < .05.

The correlation between participants’ responses to the question “Would you benefit from a generic MT training?” and to the statement “It is useful if the instructor teaches explicitly the MT aspect of the activity” was statistically significant (*r* = .22; *p* = .033). This suggests that the more people believe that they would benefit from generic MT training, the more likely they are to believe that explicit instructions regarding how to learn MT from the trainer would be helpful to improve MT learning outcomes.

A strong negative correlation (*r* = -.49; *p* < .001) was found between participants’ responses to the statement “I prefer to first learn each task on its own until I master them” and to the statement “I think the best strategy depends on the nature of the task” (Table 4). This correlation indicates that those who prefer learning in ST regime do not believe that the regime of learning depends on the task. The more participants preferred to learn using an MT regime (as expressed by lower scores on “first learn each task on its own”), the more differentiated their view on “it depends on the task”. This shows that even those who preferred MT learning may be aware that it might not be the best approach for all situations.

**Table 4:** Questions assessing participants’ opinions regarding MT learning: Correlation matrix.

		I prefer to start right away learning all tasks together	I prefer to first learn each task on its own until I master them all	It is useful if instructor teaches explicitly MT aspect of the activity	The best strategy depends on the nature of the task	Learning in MT regime differs from learning in ST regime	I would benefit from a generic MT training
I prefer to start right away learning all tasks together	r*	-	-0.003	0.229	0.115	0.112	-0.115
	p*	-	0.980	0.053	0.337	0.347	0.337
I prefer to first learn each task on its own until I master them all	*r*	-0.003	-	0.016	-0.495***	0.151	0.214
	*p*	0.980	-	0.893	< .001	0.205	0.070
It is useful if instructor teaches explicitly MT aspect of the activity	*r*	0.229	0.016	-	0.286	-0.042	0.217
	*p*	0.053	0.893	-	0.015	0.728	0.067
The best strategy depends on the nature of the task	*r*	0.115	-0.495	0.286*	-	-0.116	0.057
	*p*	0.337	< .001	0.015	-	0.331	0.635
Learning in MT regime differs from learning in ST regime	*r*	0.112	0.151	-0.042	-0.116	-	0.230
	*p*	0.347	0.205	0.728	0.331	-	0.052
I would benefit from a generic MT training	*r*	-0.115	0.214	0.217	0.057	0.230	-
	*p*	0.337	0.070	0.067	0.635	0.052	-

Note. * *p* < .05; *** *p* < .001; r* indicates Pearson’s *r* and p* indicates *p*-value.

## 4 Discussion

The present study investigated how people learn MT activities in everyday life and whether they are satisfied with their learning experience. We found that the majority of participants (43%) learned MT activities in Mix learning regimes, 33% of participants in ST regimes and the least used approach was learning in MT regimes (24%). Most of the participants were satisfied with their experiences of learning an MT activity. In addition, polychronicity, i.e., the propensity to engage in MT, positively correlated with a personal preference for pure MT learning regimes.

Our key finding is that the Mix regime is the most widely used and preferred learning regime for everyday tasks such as driving. Previous research using simple laboratory tasks has also shown that Mix regimes are more efficient for MT learning than ST regimes [8,30,31]. As mentioned before, in previous research the regimes which were called ‘MT learning’ were actually Mix regimes according to the definition in this paper because they consisted of a mix of ST and MT training. We are not aware of a study which has compared a pure MT training regime (with 100% MT training) with Mix and/or ST training regimes, so that, surprisingly, it is currently still unclear how efficient a pure MT training regime would be. Future research is required to answer this question. Taken together, current knowledge and our findings suggest that people and instructors commonly consider employing a Mix regime when learning complex everyday activities such as driving. However, these preferences may not inform whether the Mix regimes are the most effective approaches to learning complex MT activities, which means that further studies are needed.

It is plausible to assume that people choose and prefer a regime because it has advantages, and there are several conceivable advantages of a Mix regime over a pure MT regime in complex everyday tasks. First, in complex tasks it is likely that MT learning will be notably easier after the learner has at least understood how to perform the single tasks. In basic science, the single tasks are often very simple (e.g., respond with a left-button press if you hear a low-pitched beep and with a right-button press if you hear a high-pitched beep). Even in studies with such basic tasks, participants have a brief practice period where they can familiarise themselves with the tasks and practise each of the tasks. Given the simplicity of the tasks, this practice period often only needs to be a couple of trials or a few minutes long. But strictly speaking, one may consider this already a very basic form of a mix learning regime: First learn the single tasks until they are mastered to a basic level, and then switch to MT practice. In everyday tasks, such as driving, the single tasks are often considerably more complex, so that the familiarisation and initial practice periods to master them to a basic level is profoundly extended, and they constitute mix learning regimes. For future studies it would be interesting to test whether highly difficult component tasks would result in a preference for a mix learning regime–even when learning basic computerised laboratory tasks.

A limitation of the current study is that we did not acquire information about the exact amount of time and schedule of practicing in an ST regime and in an MT regime, for participants who reported having learned in a mix regime. For some activities, such as driving, it is likely that there is an initial period of extensive ST mode learning (e.g., practicing clutch-gas pedal interplay in a training area), which is followed by predominantly MT mode learning (e.g., driving in traffic). But it cannot be ruled out that, even after MT learning has commenced, people revert to ST mode learning to refine the skills of individual tasks (e.g., practicing more clutch-gas interplay). It might be the perceived proficiency of the single tasks which determines the point at which the learner should switch to an MT learning regime. Future studies could investigate whether single-task performance is a valid marker for predicting the switch from ST learning to MT learning.

A further limitation was that despite explicit explanations and instructions, some open-ended questions and choices of example tasks suggested that some participants may have been unclear about the conceptualisation and may have confused in particular MT and Mix regimes. Finally, it is important to point out that our data are based on self-reports of the participants and that for some participants the experiences of the MT learning may be quite a few years in the past.

A factor which may affect the optimal learning strategy is the individual preference to generally engage in MT, i.e., the level of polychronicity. Indeed, we found that higher levels of polychronicity, as assessed by the MPI, were linked to a stronger preference for MT learning regimes. Conversely, lower levels of polychronicity were tentatively linked to a stronger preference for ST learning regimes, although this correlation only approached significance (*p* = .113). This is interesting because it could imply interindividual differences which affect the optimal learning strategy. If these differences are known, they could be assessed before learning begins and training could be adjusted accordingly to optimise individual learning progress and outcomes. It is important to point out that we found a link between polychronicity, i.e., a preference to engage in multitasking, and preference for MT learning regimes, and future research is needed to test whether these preferences are important for actual MT learning success.

To expand our understanding of what contributed to the MT learning experience it is important to evaluate the role of an instructor or teacher. More than half of the participants reported having an instructor, and 82% of these participants were given explicit instructions of how to learn the MT aspects of the activity by their instructors. While we found that most of the participants were satisfied with the approach their instructors used, it is conceivable that approaches to instruct and learn MT activities can still be optimised further.

In line with this, we found that 92% of participants believed that learning an MT activity would benefit from a learning strategy which takes the MT aspects explicitly into account. In combination with our finding that 79% of the participants said they would benefit from generic MT training, we tentatively propose that there is a need for explicit MT training.

For future studies it would be interesting to investigate the nature of the Mix learning regimes in more detail, e.g. how long participants spend in ST and MT training, how often they switch between ST and MT training, and why they switch. Furthermore, it would be interesting to expand the focus of investigated everyday multitasking activities beyond mostly driving. Somewhat related to this, one could investigate whether prior experience in MT learning is transferrable to learning of other MT activities. Finally, it could be informative to target specific occupations with high multitasking demands in their jobs, such as air traffic controllers.

## 5 Conclusion

To conclude, our findings indicate that Mix learning regimes are the most widely used and preferred approach among participants. Many participants indicated that they would benefit from MT training, so further investigation regarding the most efficient learning regimes to learn complex everyday activities is warranted. Furthermore, we found that individual characteristics such as polychronicity may influence people’s learning preferences.
